# Evaluating the Antiparasitic Activity of Novel BPZ Derivatives Against *Toxoplasma gondii*

**DOI:** 10.3390/microorganisms8081159

**Published:** 2020-07-30

**Authors:** Olivia Hatton, Lea Stitzlein, Richard W. Dudley, Robert A. Charvat

**Affiliations:** 1 Department of Biology, University of Findlay, Findlay, OH 45840, USA; hattono@findlay.edu; 2 College of Pharmacy, University of Findlay, Findlay, OH 45840, USA; stitzleinl@findlay.edu (L.S.); dudley@findlay.edu (R.W.D.)

**Keywords:** antiparasitic, bisphenol Z, drug discovery, *Toxoplasma gondii*

## Abstract

Prevalence studies revealed that one-third of the human population is chronically infected with *Toxoplasma gondii*. Presently, such infections are without medical treatment that effectively eradicates the parasite once it is in its latent form. Moreover, the therapeutics used to treat acute infections are poorly tolerated by patients and also cause the parasite to convert into long-lasting tissue cysts. Hence, there is a dire need for compounds with antiparasitic activity against all forms of *T. gondii*. This study examines the antiparasitic capacity of nine novel bisphenol Z (BPZ) derivatives to determine whether they possessed any activity that prevented *T. gondii* replication. To begin assessing the efficacy of the novel derivatives, parasites were treated with increasing concentrations of the compounds, then doubling assays and MitoTracker staining were performed. Three of the nine compounds demonstrated strong inhibitory activity, i.e., parasite replication significantly decreased with higher concentrations. Additionally, many of the treated parasites exhibited decreases in fluorescent signaling and disruption of mitochondrial morphology. These findings suggest that bisphenol Z compounds disrupt mitochondrial function to inhibit parasite replication and may provide a foundation for the development of new and effective treatment modalities against *T. gondii*.

## 1. Introduction

*Toxoplasma gondii* is a ubiquitous pathogen known for its remarkable capacity to infect nearly any nucleated cell in both animals and humans, and thereby cause significant disease, especially in immunocompromised individuals [1,2]. Globally distributed, *T. gondii* infection impacts approximately one-third of the human population, with seroprevalence rates approaching 90% in some locales [3,4]. Within the United States, toxoplasmosis is the second leading cause of death from food-borne illness, with infection rates approaching 15% [5,6]. *T. gondii* owes some of its success to its complicated life cycle, which includes both sexual and asexual stages. Occurring solely within feline intestines, sexual reproduction allows parasites to increase their genetic diversity and produce oocysts that are shed into the environment, which subsequently mature through sporulation to become infectious [7,8]. Conversely, asexual reproduction happens in a broad range of hosts and involves two major parasite forms: tachyzoites, the tissue destructive form that rapidly divides; and bradyzoites, a latent form that persists in the host for life [9,10,11]. Infections in humans occur through a myriad of transmission routes, including congenitally [12], ingestion of water/food contaminated with infectious oocysts [13], consumption of undercooked meat from animals infected with latent bradyzoite tissue cysts [14,15], as well as through tissue transplantation [16,17]. The propensity of fast replicating tachyzoites to invade critical tissues and organs such as skeletal muscle, eyes, heart, and the nervous system of infected hosts can result in multisystem destruction from unchecked replication [18]. Despite the fact that acute infections are generally self-limited and controlled by an intact immune system, the problem arises when the parasite escapes the immune response to develop into a dormant bradyzoite tissue cyst, allowing *T. gondii* to reside within the host for life. Devastating disease manifestations occur in individuals with compromised immune systems, including lymphoma patients, organ transplant recipients, and in individuals suffering from HIV/AIDS, whereby newly acquired infections or reactivation of pre-existing tissue cysts wreak havoc [19,20,21,22,23]. Additionally, owing to the immature status of the immune system in the fetus and neonates, congenital *T. gondii* infections may result in blindness, severe neurological sequelae, and even death [24]. Moreover, there is mounting evidence correlating latent toxoplasmosis to altered behavior, as well as the development and/or exacerbation of neurodegenerative diseases and sudden-onset psychoses, such as risk-taking/reckless behavior, bipolar disorder, depression, schizophrenia, and Alzheimer’s disease [25,26,27]. Thus, the vast range of susceptible hosts and multiple routes of transmission render *T. gondii* one of the most successful pathogens on Earth, posing an immense threat to human health.

Exacerbating the gravity of human *T. gondii* infections is the fact that current therapeutics against the parasite are not only limited, but also inexorably inefficient. Presently accepted treatment strategies employ pyrimethamine and sulfadiazine [28,29] to combat acute toxoplasmosis, but these medications cause serious adverse side effects, and are complicated by the fact that they are abysmally ineffective at eradicating the chronic bradyzoite cyst [30,31]. Moreover, treating acute toxoplasmosis actually induces the conversion of the tachyzoite into the latent bradyzoite cyst, which confers the parasite with an impervious cyst wall to resist further drug challenge as well as attack by the immune system [32]. Therefore, instead of curing the patient of the infection, the therapy causes the patient to become a lifelong carrier of the parasite. Finally, the emergence of drug resistance in these parasites ablates the effectiveness of current therapeutic modalities [33,34]. Thus, due to the overall dearth of potent therapies, as well as the development and proliferation of drug-resistant parasite strains, there is increasing pressure to identify new drugs that are effective against *T. gondii* while simultaneously understanding how these new drugs affect parasite cellular biology.

In this report, we undertook an approach to evaluate *de novo* synthesized compounds built on a bisphenol Z (BPZ) molecular backbone [35]. The long-term rationale of our research is that by investigating novel compounds and understanding how they influence parasite cellular biology, we may be able to develop more effective treatment regimens to combat this highly prevalent and dangerous parasitic infection. The main aim of this short paper is to report our preliminary findings concerning our assessment of the antiparasitic activity of the BPZ parent compound and eight subsequent derivatives. Our results indicate that BPZ derivatives possess the capacity to significantly inhibit intracellular parasite replication, and that this antiparasitic activity may rely on the disruption of mitochondrial functionality and biogenesis, thereby laying the groundwork for further investigations.

## 2. Materials and Methods

### 2.1. Parasite Growth and Maintenance

All *T. gondii* cultures were maintained via serial passage through confluent human foreskin fibroblast (HFF) monolayers in normal culture medium of Dulbecco’s Modified Eagle Medium (DMEM, ThermoFisher, Waltham, MA, USA) that had been supplemented with fetal bovine serum (FBS, 10%) and a 100 units penicillin/100 µg streptomycin per mL mixture. Cultures were grown in a humidified incubator at 37 °C and 5% CO_2_. For experiments, culture dishes were scraped and parasites were released from host cells by passing through a 27-gauge needle 3–5 times to ensure adequate lysing. Next, the host cell debris was removed by passing the suspension through a 3 µm pore-size membrane filter (Whatman, ThermoFisher, Waltham, MA, USA). Free parasites were then quantified using a hemocytometer in order to establish proper parasite numbers for subsequent experiments.

### 2.2. Doubling Assays

Following syringe lysing, 500 parasites were inoculated into confluent HFF monolayers in two 24-well plates and allowed to attach and invade for five hours. Prior to treatment, free and uninvaded parasites were washed away. Parasite cultures were treated with normal culture medium supplemented with the BPZ parent compound and eight BPZ derivatives at varying concentrations (5, 10, 25, and 50 µM), Atovaquone (Sigma-Aldrich, St. Louis, MO, USA) at 2.5 µM as a positive control, and dimethyl sulfoxide (DMSO) as a negative control. The parasites underwent treatment for thirty hours before being fixed and stained in accordance with the manufacturer’s instructions for the Hema 3 Stat Pack (Fisher Scientific, Waltham, MA, USA). The parasites were quantified by counting the number of tachyzoites per parasitophorous vacuole (PV) in random fields of view until 40 PVs had been counted. The data presented are the averages of experiments completed in triplicate.

### 2.3. MitoTracker Staining and Fluorescence Microscopy

To prepare for fluorescence microscopy, monolayers of HFF cells were grown onto glass coverslips in a 24-well plate. Once confluent, the cells were infected with 30 µL of syringe lysed parasites the day before the experiment. After 18 h of growth, the medium was removed and replaced with normal culture medium, supplemented as appropriate for the various experimental conditions. Treatment conditions included BPZ parent compound and eight BPZ derivatives at 50 µM, Atovaquone at 5 µM as a positive control, and DMSO as a negative control. Following treatment for 4 h, the medium for each condition was replaced with a combination of MitoTracker Red CMXRos (Life Technologies, Carlsbad, CA, USA) at 100 nm and the test compounds for 35 min at 37 °C. Subsequently, the parasites were washed three times with fresh medium for 3 min per wash. Then, the cultures were fixed with 3.5% formaldehyde in normal culture medium for 20 min at 37 °C. The formaldehyde was then removed and quenched with PBS/100 mm glycine for 5 min, followed by one wash with PBS. The host cells and parasites were permeabilized with PBS/0.2% TX100 for 10 min, followed by five washes with PBS for 5 min each. Finally, the glass coverslips were inverted onto glass slides with 3 µL of Vectashield with DAPI (Vector Laboratories, Inc., Burlingame, CA, USA) and anchored with nail polish. The slides were visualized on a Nikon Eclipse 80i fluorescence microscope, and images were captured with NIS-Elements software and processed with ImageJ software [36].

### 2.4. Statistical Analyses

To determine whether any statistically significant differences existed between the treatment conditions, a one-way ANOVA (analysis of variance) followed by a *post hoc* Tukey’s test was completed with the α value set at 0.05. All statistical calculations were performed using Minitab v. 17.3.1 software.

## 3. Results

### 3.1. Bisphenol Z and Its Derivatives Inhibit T. gondii Replication

**Figure 1:**
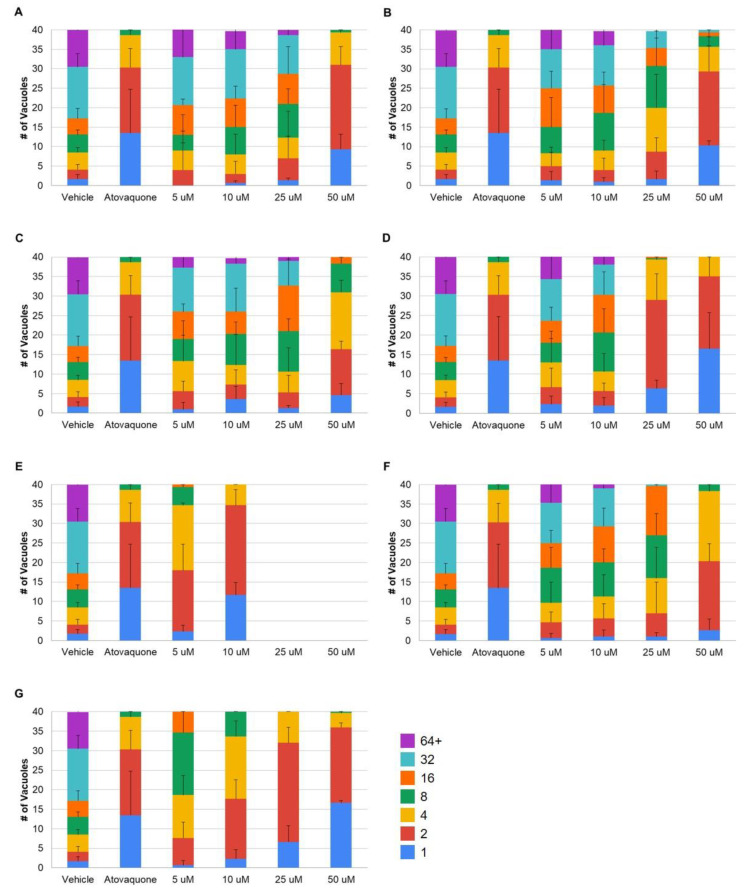
Bisphenol Z derivatives possess the capacity to inhibit the intracellular growth of *Toxoplasma gondii*, as revealed by doubling assays. Intracellular parasites were grown in the presence of increasing concentrations of the BPZ compounds for 30 h prior to fixation, staining, and enumeration of the number of parasites in each of the 40 parasitophorous vacuoles. The antiparasitic drug atovaquone (2.5 µM) was included to compare the efficacy of the (**A**) parent BPZ, (**B**) methyl-BPZ, (**C**) palmitoyl-BPZ, (**D**) ethyl-BPZ, (**E**) isopropyl-BPZ, (**F**) hexanoyl-BPZ, and (**G**) dansyl-BPZ compounds. The data presented are the averages of three independent experiments, and error bars represent standard deviation.

The cytotoxic effects of the BPZ parent compound and its derivatives were previously assessed, and it was observed that they possess an antiproliferative capacity against cancerous cell lines, including breast and glioblastoma [35]. Furthermore, it has already been reported that *T. gondii* replication can be inhibited by compounds with demonstrated anticancer properties [37]. Thus, the ability of the BPZ compounds to exert antiparasitic activity toward *T. gondii* was examined. Inhibition of parasite growth was observed for the parent BPZ compound, as well as for six of the eight tested derivatives (Figure 1), while the remaining two derivatives, i.e., amino-BPZ and hydroxyl-BPZ, displayed little to no inhibitory activity (*p* > 0.14; Table 1). Initial assessment of the parent BPZ compound revealed only a slight anti-*T. gondii* effect at 25 µM (*p* = 0.008), becoming significantly more prominent at 50 µM (*p* = 0.005; Figure 1A).

Almost all parasitophorous vacuoles (PVs) contained four or fewer tachyzoites, which is similar to the results from treatment with atovaquone (*p* = 1.00), a known antiparasitic compound [38]. For comparison, vehicle treatment with DMSO results in the majority of PVs containing 16 or more individual parasites. Likewise, equivalent results were obtained following treatment with the methyl-BPZ, palmitoyl-BPZ, and hexanoyl-BPZ derivatives (Table 1). Methyl-BPZ (Figure 1B) treatments at 25 and 50 µM were more effective at inhibiting parasite replication than treatment at 5 and 10 µM (*p* < 0.037), though not different from each other (*p* = 0.727). Parasites challenged with palmitoyl-BPZ (Figure 1C) at concentrations of 25 µM and below exhibited similar levels of growth at each concentration (*p* > 0.77), but demonstrated reduced growth compared to the vehicle treatment (*p* < 0.001). Cultures grown in 50 µM palmitoyl-BPZ were significantly more inhibited than in all other treatment conditions (*p* < 0.001), though were not statistically different than the atovaquone treatment (*p* = 0.915). The lower concentrations of hexanoyl-BPZ (5 and 10 µM) displayed equivalent antiparasitic activity (*p* = 0.659; Figure 1F). The inhibitory activity at 25 and 50 µM was augmented compared to the lower concentrations (*p* < 0.003), although the results for each of the higher concentrations were the same (*p* = 0.286).

Conversely, when *T. gondii* infected cells were treated with the ethyl-BPZ, isopropyl-BPZ, and dansyl-BPZ derivatives, parasite replication was greatly inhibited at lower concentrations (Table 1). For the ethyl-BPZ derivative, mild growth inhibition occurred at 5 and 10 µM (*p* < 0.02), which dramatically increased at 25 and 50 µM (*p* < 0.001), i.e., to such an extent that as many as 87.5% of PVs presented with two or fewer parasites at the highest concentration tested (Figure 1D). The dansyl-BPZ derivative exhibited a dose response with moderate inhibitory activity being observed at the lowest concentration tested (5 µM; *p* < 0.001), i.e., where 46.7% of vacuoles contained ≤4 tachyzoites each (Figure 1G). Though treatment at concentrations greater than 5 µM resulted in the 4-pack vacuoles exceeding a percentage of 85%, the only statistically significant difference in effectiveness was observed between the 5 and 50 µM concentrations (*p* = 0.043). The greatest anti-*T. gondii* activity was observed following treatment with the isopropyl-BPZ derivative (Table 1). Tremendous inhibition of parasite replication was recorded at both 5 and 10 µM (*p* < 0.001), with 45.0% and 86.7% of PVs possessing two or fewer parasites, respectively (Figure 1E), while there was no discernible difference between these concentrations and atovaquone (*p* > 0.74). Although host cell viability began to be impacted (based on our observations as well as personal communication with R.D.) at concentrations above 10 µM, not a single parasitophorous vacuole was observed to contain more than one individual parasite (data not shown), indicating that *T. gondii* replication was completely prevented. Taken together, these findings suggest that bisphenol Z and the majority of its derivatives possess antiparasitic activity against *T. gondii*, warranting further studies to explore the inhibitory effects of BPZ.

### 3.2. The Parasite Mitochondrion Is Impacted by BPZ and Its Derivatives

**Figure 2:**
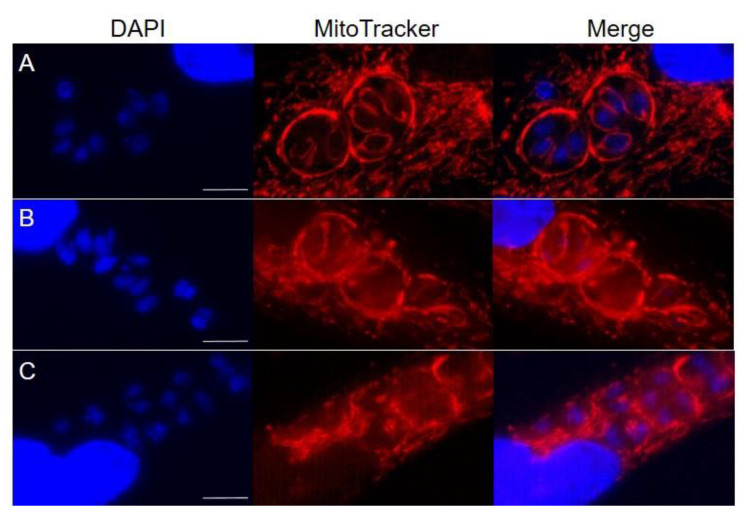
Mitochondrial membrane potential and morphology were disrupted in the presence of the BPZ derivatives. Intracellular parasites were grown in the presence of (**A**) Dimethyl sulfoxide (DMSO) (**B**) bisphenol Z (BPZ) (50 µM), or (**C**) isopropyl-BPZ (50 µM) for 4 h prior to MitoTracker staining, fixation, and imaging. Scale bars represent 5 µm.

A number of studies have demonstrated that the antiparasitic effectiveness of compounds used as cancer chemotherapeutics stems from an alteration of mitochondrial biogenesis and function, including ultrastructural perturbations of the mitochondrial matrix [39], as well as disrupted redox homeostasis that results in the generation of reactive oxygen species [40,41]. As a means to initially assess the cellular consequences of the challenge with the BPZ derivatives against *T. gondii*, we examined the fluorescent signal of parasites stained with MitoTracker in order to assess the mitochondrial membrane potential. As shown in Figure 2A, parasite cultures treated with the DMSO vehicle control displayed brightly stained host and parasite mitochondria. Moreover, the classic lasso appearance of the sole parasite mitochondrion was distinctly observed [42]. Following treatment with the BPZ parent compound, it was noted that the MitoTracker staining became less distinct in the mitochondrion and more perfuse throughout the PV (Figure 2B). Additionally, it was observed that some PVs presented with fewer lasso morphologies. Finally, when parasite cultures were treated with the isopropyl-BPZ derivative, i.e., the compound exhibiting the greatest anti-*T. gondii* activity in doubling assays, MitoTracker staining was greatly reduced in parasite mitochondria, and the morphological appearance of those mitochondria became more punctate in structure (Figure 2C). All results considered, the decreased staining intensity with MitoTracker combined with a more diffuse fluorescent signal in the PVs, as well as the disrupted mitochondrial morphology, offer a glimpse into the potential mechanism of action for the growth inhibitory activity of these novel BPZ compounds.

## 4. Discussion

*Toxoplasma gondii* is a ubiquitous parasite and a significant cause of morbidity and mortality, making it a subject of concern in both veterinary and human medicine. Often touted as one of the most successful parasites on Earth, *T. gondii*’s attainment of such a moniker is due to its high global prevalence, exceedingly broad host range, multiple routes of transmission, and conversion into an impervious tissue cyst that persists in the host for life. Reinforcing its world dominance is the fact that current treatment strategies are lacking in both effectiveness and tolerability in patients. Considering all the unique facets of *T. gondii* cell biology, disease manifestation, and therapeutic shortcomings, significant strides must be made toward developing and improving modalities for eradicating this parasite and alleviating patient suffering.

Working toward the aforementioned goal, this brief report is the first to report the antiparasitic activity of a bisphenol Z compound and novel derivatives thereof. Our preliminary observations allude to impacts on the parasite mitochondrion as a precursor stimulus to the cytotoxic effects and growth inhibition of *T. gondii*. Examination of the ability of the BPZ compounds to disrupt mitochondrial membrane potential revealed decreased MitoTracker staining as well as alterations in overall mitochondrial morphology. Seemingly, these effects culminated in the inability of the parasite to replicate beyond one or two divisions in the presence of the BPZ derivatives, whereas abundant growth was observed in vehicle treated parasites. These findings provide the foundation for continued evaluation of the anti-*T. gondii* efficacy of bisphenol Z derivatives.

In considering the potential targets of the BPZ compounds, we hypothesize that the mitochondrion, being a critical organelle for normal parasite biology, is an excellent target for drug activity, in order to cause growth inhibition and eventual parasite death. This is unsurprising as the mitochondrion plays an important role in providing energy to the cell, as well as regulating the balance between life and death signals. Additionally, the *T. gondii* mitochondrion appears to serve a critical function as a sentinel for the detection of genotoxic assaults on the cell [43]. Perhaps one of the features of *T. gondii*—as well as of other pathogenic protozoan parasites—that provides abundant opportunities for the development of antiparasitic compounds is that these parasites possess only one of these vital organelles [44,45,46]. Indeed, a number of previous studies have reported that the antiparasitic effectiveness of their tested compounds appeared to converge on the parasite mitochondrion as a part of the mechanism of action [38,39,40,41,47,48,49,50,51,52,53]. Furthermore, unlike many higher eukaryotes that possess abundant mitochondria to maintain an overall healthy organelle pool [54], the fact that these parasites contain a single mitochondrion suggests that they might lack the critical homeostatic mechanisms for mitochondrial maintenance. Such a vulnerability has prompted several groups to undertake research endeavors to identify the proteins and pathways involved in the biogenesis of the mitochondrion [55,56,57,58]. Likewise, our own upcoming research endeavors are focusing on the identification of the cellular target(s) of the BPZ compounds to illuminate their mechanism of action in the hope of spurring on the development of new treatment regimens against such critical processes.

Future research directions will involve gaining a deeper understanding of the inhibitory activity of the BPZ derivatives, including examining all steps involved in the *T. gondii* lytic replication cycle. An interesting observation that we made, but one which would require validation, is the fact that not all of the BPZ derivatives were capable of inhibiting the intracellular replication of the parasites. For example, amino-BPZ and hydroxyl-BPZ exhibited no activity in the doubling assays, but in a proof-of-concept experiment, we noticed that these derivatives reduced the formation of parasite plaques. If corroborated, this finding would suggest that certain BPZ compounds inhibit endodyogeny [59], the process by which *T. gondii* divides, while others prevent additional stages of the lytic cycle from occurring which are critical for the progression of an infection, namely attachment, entry, or egress [60]. Validation of BPZ derivatives possessing divergent inhibitory activity could establish a basis for a combinatorial approach to blocking multiple processes in the parasite to combat this ever-present pathogen.

## Figures and Tables

**Table 1:** Average number of *T. gondii* tachyzoites per vacuole.

Treatment	5 µM	10 µM	25 µM	50 µM
DMSO ^1^	28.84			
atovaquone ^2^	2.28			
BPZ	26.17	22.57	15.78	2.28
amino-BPZ	22.95	24.21	22.28	16.42
methyl-BPZ	21.88	19.54	8.99	3.31
palmitoyl-BPZ	18.29	16.64	14.17	4.30
ethyl-BPZ	21.78	15.93	2.53	1.84
isopropyl-BPZ	3.71	1.98	- ^3^	-
hexanoyl-BPZ	20.78	15.63	8.76	3.08
hydroxyl-BPZ	25.58	20.34	22.50	20.87
dansyl-BPZ	6.80	3.69	2.23	1.82

^1^ Dimethyl sulfoxide (DMSO) volume equivalent to the volume used at 50 µM for bisphenol Z (BPZ) derivatives. ^2^ Atovaquone concentration used was 2.5 µM. ^3^ Host cell cytotoxicity observed.
